# Trends and Patterns of Antibiotics Use in China’s Urban Tertiary Hospitals, 2016–19

**DOI:** 10.3389/fphar.2021.757309

**Published:** 2021-11-03

**Authors:** Yulei Zhu, Yang Qiao, Rouli Dai, Xin Hu, Xin Li

**Affiliations:** ^1^ Office of Scientific Research, the Affiliated Stomatological Hospital of Nanjing Medical University, Nanjing, China; ^2^ School of Health Policy and Management, Nanjing Medical University, Nanjing, China; ^3^ First School of Clinical Medicine, Nanjing Medical University, Nanjing, China; ^4^ Medical Office, The Fourth Affiliated Hospital of Nanjing Medical University, Nanjing, China; ^5^ Department of Pharmacy, Beijing Hospital, National Center of Gerontology, Institute of Geriatric Medicine, Chinese Academy of Medical Sciences, Beijing Key Laboratory of Assessment of Clinical Drugs Risk and Individual Application (Beijing Hospital), Beijing, China; ^6^ Department of Clinical Pharmacy, School of Pharmacy, Nanjing Medical University, Nanjing, China; ^7^ Center for Global Health, School of Public Health, Nanjing Medical University, Nanjing, China

**Keywords:** antimicrobial stewardship, antibiotics use, tertiary hospitals, urban hospitals, China

## Abstract

**Objectives:** This study aimed to identify the trends in antibiotics utilization and patients costs, evaluating the effect of the policy and exploring factors associated with the irrational use of antibiotics.

**Methods:** Based on the Cooperation Project Database of Hospital Prescriptions, data were collected from 89 tertiary hospitals in nine cities in China during 2016–2019. The study sample consisted of prescription records with antibiotics for 3,422,710 outpatient and emergency visits and 26, 118, 436 inpatient hospitalizations.

**Results:** For outpatients, the proportion of treated with antibiotics declined from 14.72 to 13.92% significantly (*p* < 0.01). The proportion of antibiotic costs for outpatients decreased from 5.79 to 4.45% significantly (*p* < 0.01). For emergency patients, the proportion of treated with antibiotics increased from 39.31 to 43.45% significantly (*p* < 0.01). The proportion of antibiotic costs for emergency patients decreased from 36.44 to 34.69%, with no significant change (*p* = 0.87). For inpatients, the proportion of treated with antibiotics increased from 23.82 to 27.25% significantly (*p* < 0.01). The proportion of antibiotic costs for outpatients decreased from 18.09 to 17.19% with no statistical significance (*p* = 0.89). Other β-lactam antibacterials (1,663.03 ten thousand DDD) far exceeded other antibiotics categories. Stablely ranked first, followed by Macrolides, lincosamide and streptogramins (965.74 ten thousand DDD), Quinolone antibacterials (710.42 ten thousand DDD), and β-lactam antibacterials, penicillins (497.01 ten thousand DDD).

**Conclusions:** The proportion of treated with antibiotics for outpatients and inpatients meet the WHO standards. The antibiotics use varied by different survey areas, clinical departments, patient gender, patient age and antibiotics categories. More efforts should focus on improving the appropriateness of antibiotics use at the individual level.

## Introduction

Antibiotics are medicinal products that kill or inhibit the growth of living microorganisms (European Centre for Disease Prevention and Control, 2018). As one of the most important discoveries in pharmaceuticals, antibiotics have saved millions of lives worldwide. However, when antibiotics are used the emergence of antimicrobial resistance (AMR) is inevitable. Statistically, the consumption of antibiotics increased by 36% from 2000 to 2010 (Van Boeckel et al., 2014). Meanwhile, the number of new antibiotics approved for the market worldwide is decreasing (Liu, 2018). AMR is regarded as one of the greatest threats to global health by the World Health Organization (WHO) (World Health Organization, 2015), which has the potential to affect anyone, of any age, in any country (World Health Organization, 2018). We have now reached a crisis where many antimicrobials are no longer effective against previously curable infectious diseases. As well as the emergence of AMR, prolonged hospitalization, more substantial social and economic burden, increased human suffering, and more premature deaths are the results of inappropriate antibiotics (Gandra et al., 2014; Li et al., 2019). According to a review chaired by the English Economist JIM O’NEILL, if AMR is not curtailed, 10 million people will die of drug-resistant bacterial infection every year by 2050 worldwide, with cumulative economic loss up to 100 trillion USD in total (Jim, 2015). In 2015, the WHO Global Action Plan on AMR was adopted by the member states at the 68th World Health Assembly, which aimed to promote the use of antibiotics (World Health Organization, 2016).

China is one of the largest consumers of antibiotics globally (Van Boeckel et al., 2014), and the abuse of antibiotics is relatively severe in this country (Yin et al., 2013; Wang et al., 2014). In an attempt to optimize the appropriate use of antimicrobial agents, the Chinese government has issued a series of policies and regulations in the last few years. In 2011, China initiated the National Special Rectification Campaign (NSRC) by its ministry of health targeting AMR and rational use of antimicrobial agents at secondary and tertiary hospitals (Chinese Ministry of Health, 2011), which achieved significant effects after several years of implementation (Bao et al., 2015; Xiao, 2018). On August 1, 2012, the strictest regulation yet for antimicrobial stewardship in China was formally taken into effect, which clearly defined all aspects of antibiotic use in health-care institutions (Xiao and Li, 2013). To ensure sustainable progress towards antibiotics’ rational use, China also launched its National Action Plan to Curve Antimicrobial Resistance (2016–2020) in 2016. The critical intervention of the action plan for health-care institutions, including 1) to standardize management of the use of antibacterial agents; 2) to optimize the AMR surveillance network in clinics; 3) to enhance the training of professional personnel in the rational use of antibiotics and AMR (National Health and Family Plan Commission of China, 2016; Xiao and Li, 2016).

Although there have been many studies of antibiotic use worldwide, a few studies were multicenter based on continuous data, especially in China. Furthermore, only a few studies on antibiotic prescribing patterns in health-care institutions have been conducted in China, using national or provincial data. Of these, two studies surveys focused on antibiotic prescribing patterns in primary health-care institutions before 2012 (Dong et al., 2008; Wang et al., 2014); one analyzed antibiotic use in specialized hospitals during 2011–12 (Zou et al., 2014), and two focused on antibiotic prescribing and costs in secondary and tertiary health-care institutions before 2016 (Bao et al., 2015; Li et al., 2019). Bao et al. conducted a comparative analysis of antimicrobial use in 65 general public hospitals in China, which found that the National Action Plan for antibiotic stewardship targeting antibiotic misuse had a positive effect in reducing antibiotic consumption and expenditure (Bao et al., 2015). A study based on the Center for Antibacterial Surveillance also demonstrated a significant downward trend in antibiotic use in general hospitals in two provinces of China after implementing restriction. The policy intervention might optimize the use of the antibiotic (Li et al., 2019).

None of these studies conducted a comparative analysis of antibiotic use after implementing the strictest regulation in 2016. Further research is required to identify the effectiveness of policy interventions in China. Former studies demonstrated that most of the antibiotics were procured and consumed by secondary and tertiary hospitals, especially in tertiary hospitals (Lin et al., 2016; Yin et al., 2018). Besides, a few studies focused on comparable antibiotic use data at a population level in tertiary hospitals. Thus, based on the retrospective data from 89 tertiary hospitals in nine cities in China during 2016–2019. We focused on identifying the trends in antibiotics utilization and patients costs, evaluating the effect of the policy and exploring factors associated with the irrational use of antibiotics. We expected that this study’s findings could provide a basis for future antibiotic stewardship by policy-makers and health-care institutions.

## Methods

### Data Sources

**FIGURE 1:**
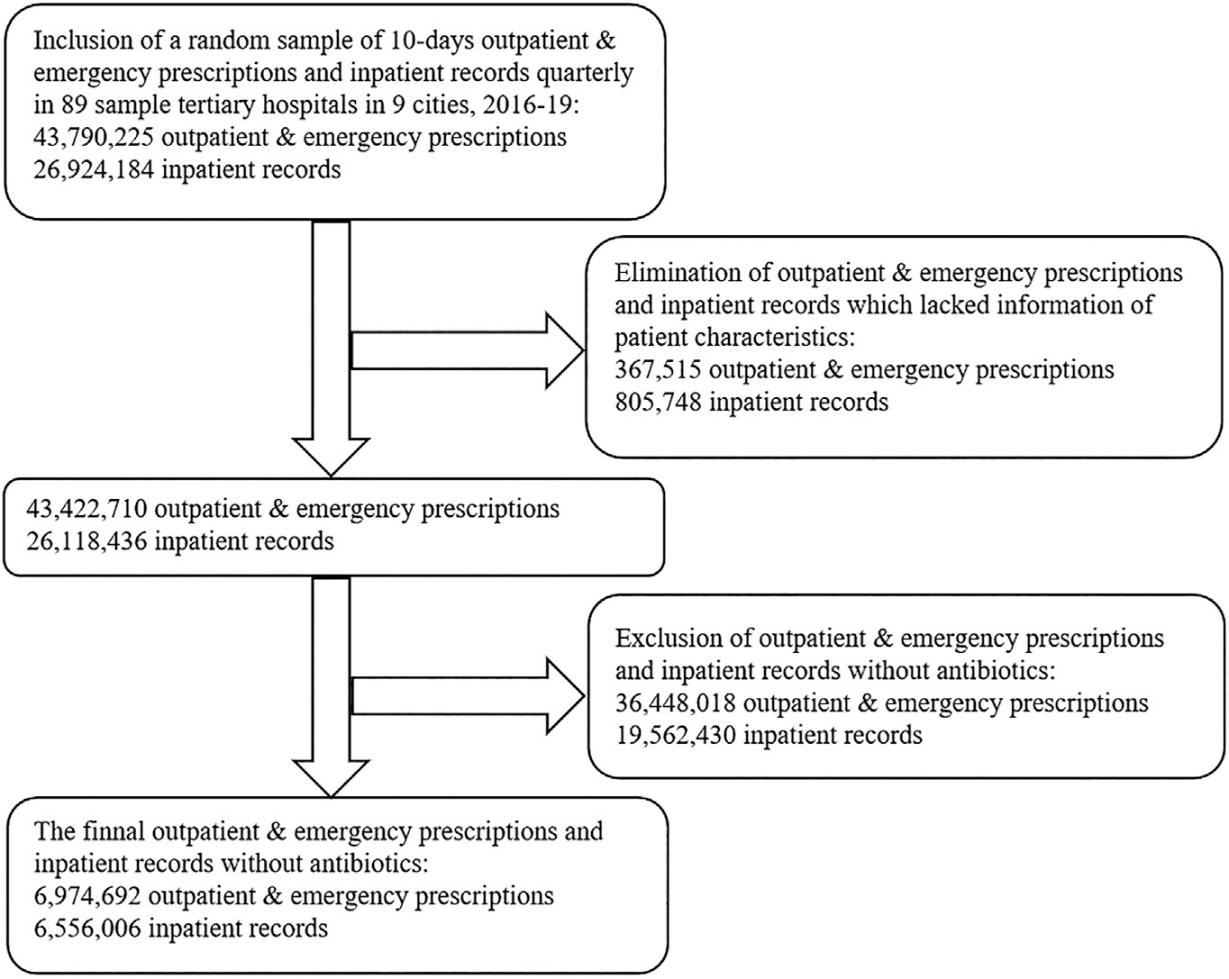
Flow diagram showing the process of selecting prescriptions and records with antibiotics based on the Cooperation Project Database of Hospital Prescriptions.

A convenient sampling method was used in this study included 89 tertiary hospitals in China. Information pertaining to the use of antibiotics, including sample city, patient characteristic (gender and age), visit time, type of care (Outpatient/Emergency/Inpatient), clinical department, the number of all Outpatient/Emergency prescriptions or inpatient records with antibiotics, prescribed antibiotics (dosage form, strength, packaging specifications, pharmaceutical manufacturer, and cost) and additional information (the number of all prescriptions/records on each day of sampling, the cost of all pharmaceuticals on each day of sampling) was gathered from the Cooperation Project Database of Hospital Prescriptions (CPDHP). The CPDHP is an extensive database with medicine records procurement covering public tertiary hospitals in nine cities, China (including Beijing, Chengdu, Guangzhou, Hangzhou, Harbin, Shanghai, Shenyang, Tianjin and Zhengzhou). 44.44% of cities total gross domestic product (GDP) was over 15 ten thousand RMB (∼21.8 thousand USD) in 2019, and 33.33% was below 10 ten thousand RMB (∼14.5 thousand USD). A total of 89 tertiary hospitals were included in the current study. Beijing accounted for the largest number of survey hospitals (13 out of 89), and Tianjing was the lowest (6 of 89). The value of Gross Domestic Product varied among the different survey cities, which was highest in Beijing (16.40 ten thousand yuan in 2019) and lowest in Harbin (5.52 ten thousand yuan in 2019). We included the clinical data from the first quarter of 2016 (2016Q1) to the fourth quarter of 2019 (2019Q4). A random sample of 10-days outpatient and emergency prescriptions and inpatient records quarterly in 89 sample tertiary hospitals in nine cities, 2016–19, were aggregated and standardized by engineers for research and decision-making. The events without complete information were excluded, as well as prescription/records without antibiotics (Figure 1).

All antibiotics prescribed were coded using Anatomical Therapeutic Chemical (ATC) classification system, which was divided into subgroups: Tetracyclines (J01A); *β*-lactam antibacterials, penicillins (J01C); Other *β*-lactam antibacterials (J01D); Macrolides, lincosamide and streptogramins (J01F); Quinolone antibacterials (J01M); Other antibiotics in J01; and antimycotics for systemic use (J02A). According to the guidelines for clinical governance, the antibiotics were categorized into three categories: non-restricted, restricted and controlled antibiotics. In the current study, we focused on systemic agents per oral or parenteral administration route and did not include topical agents such as skin creams, ophthalmic ointments, etc.

### Indicators and Definitions

The significant indicators were selected in accordance with the standardized method of rational drug use developed by the WHO and International Network (WHO, 1993). Antibiotics prescribing was analyzed using seven indicators as follows:

1)Proportion of treated with antibiotics = numbers of prescriptions or records that included at least one antibiotic/total numbers of prescriptions or records × 100%;
2)Proportion of prescribed with muti-antibiotics = numbers of prescriptions or records that included greater than or equal to two antibiotics with different ATC codes/numbers of prescriptions or records that included at least one antibiotic × 100%;
3)Proportion of prescribed with parenteral antibiotics = numbers of prescriptions or records that included at least one injection/numbers of prescriptions or records that included at least one antibiotic × 100%;
4)Average drugs expenditure per visit = total drug expenditure/numbers of prescriptions or records;
5)Average antibiotics expenditure per visit = antibiotic expenditure/numbers of prescriptions or records that included at least one antibiotic;
6)Proportion of antibiotics expenditure = antibiotic expenditure/total drug expenditure;
7)Defined daily doses (DDDs) of antibiotics = total volume of medicine consumed (i)/DDD_i_ (DDD_i_ is a unit of measure that refers to the average maintenance dosage of each medicine used for its main indication in adults daily, which was obtained from the WHO DDD index web page) (The WHO Collaborating Centre for Drug Statistics Methodology, 2019).

### Statistical Analysis

The quantitative data were analyzed by mean value each quarter to assess trends in the overall number or rate of indicators during the study period. We used the Cochran-Armitage trend test was used to examine the trend of categorical variables over time and linear regression analysis was applied to test the trend of continuous variables over time. The Cochran-Armitage test emphasizes the dichotomous data of occurrence, which can be applied to evaluate the trend in the rates such as disease incidence, symptom Occurrence and etc (Bieler and Williams, 1993; Wang et al., 2017). Besides, the test was performed in one survey focused on the trend in antibiotics prescription rates in general hospitals in two provinces of China (Li et al., 2019). In this study, one of the classified variables is whether a prescription contained antibiotics or not, and the other variable is quarter. R 4.0.4 and IBM SPSS (version 22.0; Chicago, IL, United States) was used to perform the above analysis, and the *p*-value <0.05 was considered statistically significant.

## Results

### General Information of the Sampled Prescriptions/Records With Antibiotics

In the nine cities selected, we collected all 89 public hospitals prescribing data for 4 years consecutively. A total of 5,601,377 outpatient prescriptions, 1,527,034 emergency prescriptions and 6,402,287 inpatient records included at least one antibiotic were obtained in the current study. Among them, 50.74% were male patients, and 49.26% were female patients. Among 13, 530, 698 sample data, 35.06% were patients between the age of 18 and 49 years old. Internal medicine (24.15%) and Surgery (23.09%) were the top two clinical departments where patients received prescriptions containing antibiotics. The details of the annual data are shown in Table 1.

**TABLE 1:** Patient-related associated with antibiotic prescribing in health-care institutions in nine cities, 2016–19.

Characteristics	2016	2017	2018	2019
Total antibiotic-containing prescriptions(n)	3,162,377	3,315,731	3,411,229	3,641,361
Patient gender	—	—	—	—
Male	1,602,436 (50.67)	1,681,661 (50.72)	1,735,374 (50.87)	1,846,230 (50.70)
Female	1,559,941 (49.33)	1,634,070 (49.28)	1,675,855 (49.13)	1,795,131 (49.30)
Patient age	—	—	—	—
<5 years	251,576 (7.96)	264,135 (7.97)	263,248 (7.72)	278,383 (7.65)
5–17 years	213,394 (6.75)	223,776 (6.75)	221,732 (6.50)	265,223 (7.28)
18–49 years	1,121,451 (35.46)	1,161,607 (35.03)	1,193,072 (34.97)	1,267,680 (34.81)
50–64 years	725,602 (22.94)	753,637 (22.73)	784,684 (23.00)	819,011 (22.49)
≥65 years	850,354 (26.89)	912,576 (27.52)	948,493 (27.81)	1,011,064 (27.77)
Care type	—	—	—	—
Outpatient	1,347,105 (42.60)	1,358,617 (40.97)	1,395,468 (40.91)	1,500,187 (41.20)
Emergency	318,918 (10.08)	368,358 (11.11)	392,639 (11.51)	447,119 (12.28)
Inpatient	1,496,354 (47.32)	1,588,756 (47.92)	1,623,122 (47.58)	1,694,055 (46.52)
Clinical department	—	—	—	—
Emergency internal medicine	263,085 (8.32)	307,436 (9.27)	329,825 (9.67)	374,524 (10.29)
Emergency surgery	55,833 (1.77)	60,922 (1.84)	62,814 (1.84)	72,595 (1.99)
Internal medicine	787,401 (24.90)	800,498 (24.14)	819,174 (24.01)	860,962 (23.64)
Surgery	731,658 (23.14)	768,100 (23.17)	795,327 (23.31)	829,376 (22.78)
Stomatology	100,235 (3.17)	111,921 (3.38)	113,132 (3.32)	117,115 (3.22)
Respiratory medicine	347,636 (10.99)	356,565 (10.75)	372,205 (10.91)	391,078 (10.74)
Infectious disease	81,314 (2.57)	93,313 (2.81)	96,588 (2.83)	112,126 (3.08)
Obstetrics and gynecology	212,919 (6.73)	216,299 (6.52)	211,487 (6.20)	210,909 (5.79)
Pediatrics	365,991 (11.57)	383,804 (11.58)	380,064 (11.14)	431,723 (11.86)
ICU	56,938 (1.80)	59,688 (1.80)	61,880 (1.81)	65,569 (1.80)
Other	159,367 (5.04)	157,185 (4.74)	168,733 (4.95)	175,384 (4.82)
Survey region	—	—	—	—
Beijing	380,342 (12.03)	375,927 (11.34)	380,944 (11.17)	398,830 (10.95)
Chengdu	517,728 (16.37)	530,832 (16.01)	515,394 (15.11)	533,951 (14.66)
Shenyang	194,648 (6.16)	199,419 (6.01)	205,098 (6.01)	213,693 (5.87)
Tianjin	199,067 (6.29)	233,805 (7.05)	250,094 (7.33)	269,587 (7.40)
Zhengzhou	194,049 (6.14)	204,359 (6.16)	218,265 (6.40)	230,066 (6.32)
Guangzhou	570,311 (18.03)	579,717 (17.48)	604,075 (17.71)	659,371 (18.11)
Hangzhou	430,645 (13.62)	467,655 (14.10)	519,215 (15.22)	566,629 (15.56)
Harbin	182,713 (5.78)	190,673 (5.75)	179,559 (5.26)	200,107 (5.50)
Shanghai	492,874 (15.59)	533,344 (16.09)	538,585 (15.79)	569,127 (15.63)

Unless otherwise indicated, values shown are n(%).

### Overall Pharmaceuticals Consumption

**FIGURE 2:**
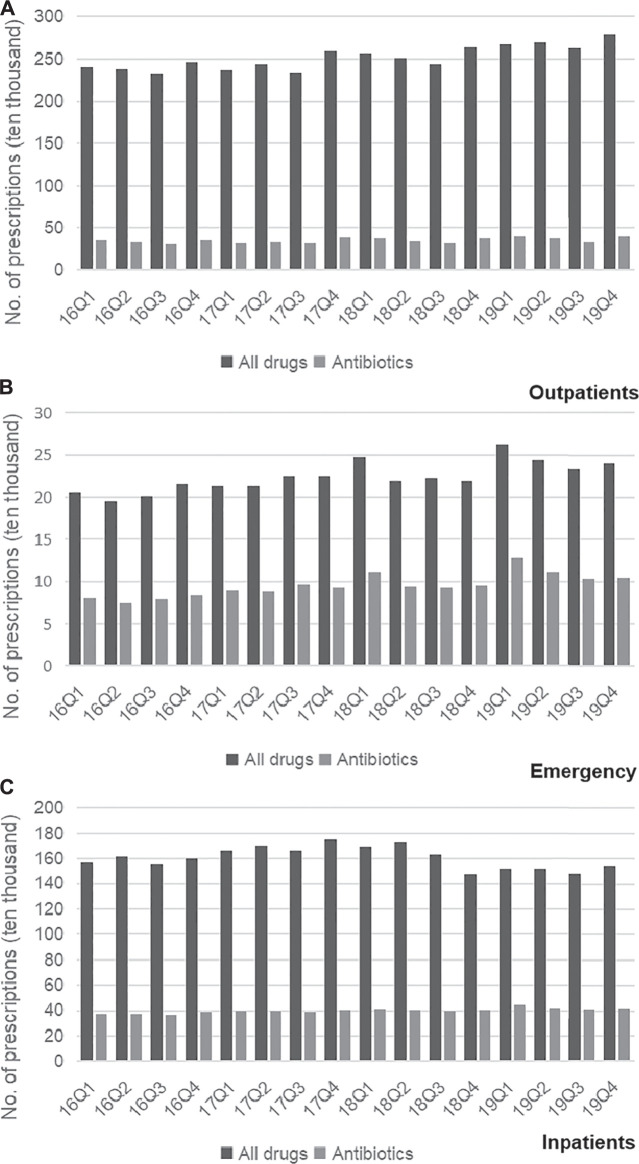
**(A)** Changes in prescriptions of drugs in Outpatients, 2016–19. **(B)** Changes in prescriptions of drugs in Emergency, 2016–19. **(C)** Changes in prescriptions of drugs in Inpatients, 2016–19.

**FIGURE 3:**
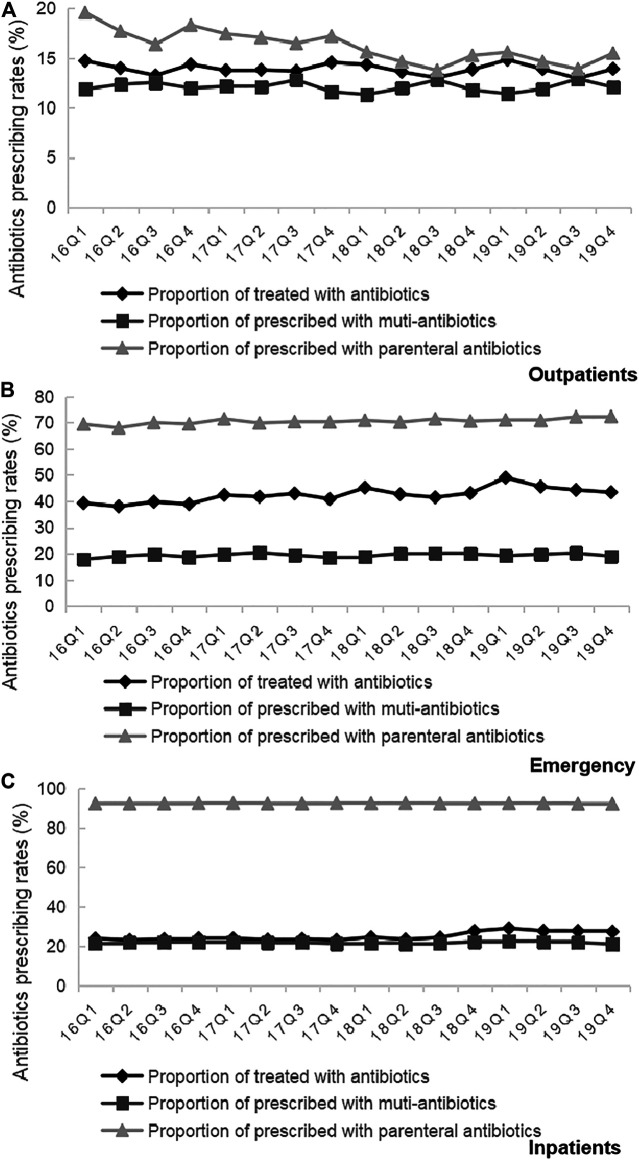
**(A)** Changes in antibiotics prescribing rates in Outpatients, 2016–19. **(B)** Changes in antibiotics prescribing rates in Emergency, 2016–19. **(C)** Changes in antibiotics prescribing rates in Inpatients, 2016–19.

**FIGURE 4:**
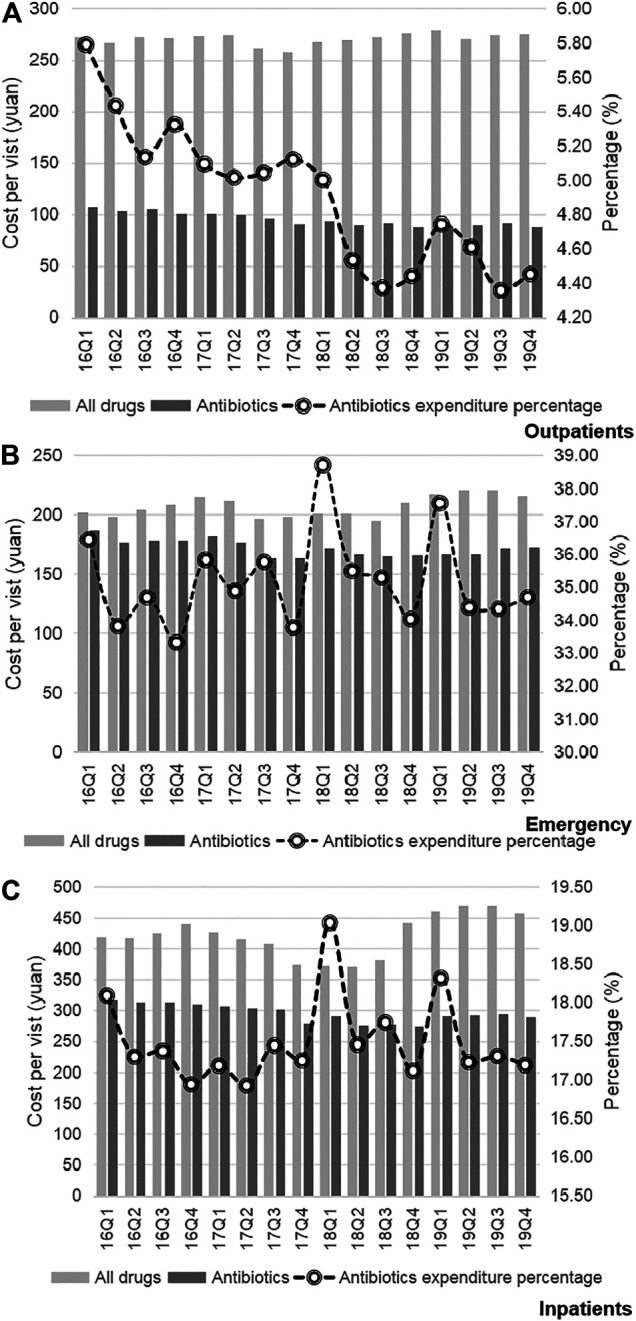
**(A)** Changes in antibiotics expenditure in Outpatients, 2016–19. **(B)** Changes in antibiotics expenditure in Emergency, 2016–19. **(C)** Changes in antibiotics expenditure in Inpatients, 2016–19.

Trends in the usage of pharmaceuticals are shown in Figure 2 and Figure 3. The average amount of all drugs prescriptions for outpatients was 251.43 ten thousand, and it increased significantly throughout the study period (*p* < 0.01); the average amount of prescriptions containing antibiotics was 35.01 ten thousand, and it increased significantly throughout the study period (*p* < 0.05). In contrast, the average proportion of treated with antibiotics and proportion of prescribed with muti-antibiotics for outpatients was 13.92 and 12.21%, respectively, which dropped from 2016Q1 to 2019Q4 (*p* < 0.01). The average amount of all drugs prescriptions for patients of emergency department was 22.39 ten thousand, and it increased significantly throughout the study period (*p* < 0.01); the average amount of prescriptions containing antibiotics was 9.54 ten thousand, and it increased significantly throughout the study period (*p* < 0.01). The average proportion of treated with antibiotics and proportion of prescribed with parenteral antibiotics in emergency department was 42.44 and 70.50%, respectively, which increased from 2016Q1 to 2019Q4 (*p* < 0.01). For inpatients, the number of all drugs records was 163.24 ten thousand on average, with no significant changes during the study period (*p* = 0.14); the average amount of records containing antibiotics was 40.98 ten thousand, and it increased significantly throughout the study period (*p* < 0.01). The proportion of treated with antibiotics was 25.19% on average, showing significant uptrend over the 16 quarters (*p* < 0.01). Figure 4 shows the changes in antibiotics expenditure. As for the expenditure of the drugs, the cost of antibiotics for outpatients increased from 201.91 yuan to 216.11 yuan (*p* < 0.01), and the proportion of antibiotics cost dropped from 5.79 to 4.45% (*p* < 0.05); but there was no significant change in the cost of all drugs (*p* = 0.31). The cost of all drugs in emergency departments increased from 201.91 yuan to 216.11 yuan (*p* < 0.05), and the cost of antibiotics cost in dropped from 187.18 yuan to 172.56 yuan (*p* < 0.01); but there was no significant change in the proportion of antibiotics cost (*p* = 0.31). For inpatients, the cost of antibiotics showed a linear downward trend over time, with a decrease of 30.09 yuan (*p* < 0.01), but no significant change was observed in the cost of all drugs (*p* = 0.14) and the proportion of antibiotics cost (*p* = 0.75).

### Antibiotics Prescribing Patterns

Table 2 shows the quarterly trend in the proportion of antibiotics from 2016 to 2019 in different clinical departments. For outpatients, the proportion of treated with antibiotics in Infectious disease (*p* < 0.01) and Other (*p* < 0.01) increased over time; the proportion of treated with antibiotics in Obstetrics and gynecology (*p* < 0.01), Respiratory medicine (*p* < 0.01), Stomatology (*p* < 0.01), Internal medicine (*p* < 0.01) and Surgery (*p* < 0.01) were opposite. For patients in emergency departments, there was an uptrend in the proportion of treated with antibiotics in the Emergency internal medicine (*p* < 0.01) and Emergency surgery (*p* < 0.01). For inpatients, the proportion of treated with antibiotics in the Pediatrics (*p* < 0.01), Obstetrics and gynecology (*p* < 0.01), Infectious disease (*p* < 0.01), Respiratory medicine (*p* < 0.01), Stomatology (*p* < 0.01), Internal medicine (*p* < 0.01), Surgery (*p* < 0.01), ICU (*p* < 0.01) and Other (*p* < 0.01) increased from 2016Q1 to 2019Q4. In terms of the muti-antibiotics usage, the percentage in Internal medicine (*p* < 0.01) for outpatients increased from 2016Q1 to 2019Q4, which in contrast to the percentage in Pediatrics (*p* < 0.01), Infectious disease (*p* < 0.01), Respiratory medicine (*p* < 0.01), Surgery (*p* < 0.01) and Other (*p* < 0.01). For patients in emergency departments, there was an uptrend in the proportion of muti-antibiotics in the Emergency internal medicine (*p* < 0.01), but the proportion of muti-antibiotics in Emergency surgery was opposite (*p* < 0.01). For inpatients, the proportion of prescribed with muti-antibiotics in the Pediatrics (*p* < 0.01), Obstetrics and gynecology (*p* < 0.01), Respiratory medicine (*p* < 0.01), Internal medicine (*p* < 0.01), ICU (*p* < 0.01) and Other (*p* < 0.01) increased over time, but the proportion of prescribed with muti-antibiotics in Surgery (*p* < 0.01) was opposite (Table 3). In terms of the parenteral antibiotics usage, the percentage in Pediatrics (*p* < 0.01), Obstetrics and gynecology (*p* < 0.01), Respiratory medicine (*p* < 0.01), Internal medicine (*p* < 0.01) and Surgery (*p* < 0.01) for outpatients dropped from 2016Q1 to 2019Q4. For patients in emergency departments, there was an uptrend in the proportion of prescribed with parenteral antibiotics in the Emergency internal medicine (*p* < 0.01) and Emergency surgery (*p* < 0.01). For inpatients, the proportion of prescribed with parenteral antibiotics in Obstetrics and gynecology (*p* < 0.01) increased over time; the proportion of prescribed with parenteral antibiotics in Infectious disease (*p* < 0.01), Respiratory medicine (*p* < 0.01), and Other (*p* < 0.01) (Table 4).

**TABLE 2:** Changes in proportion of treated with antibiotics in different clinical departments, 2016–19.

Care type	Clinical department	16Q1	16Q2	16Q3	16Q4	17Q1	17Q2	17Q3	17Q4	18Q1	18Q2	18Q3	18Q4	19Q1	19Q2	19Q3	19Q4	Z^a^
**Outpatient prescriptions**	
	Pediatrics	45.96	46.08	41.8	48.97	44.58	46.92	42	47.67	43.16	47.26	42.17	47.81	44.14	46.52	43.41	46.47	−1.511
	Obstetrics and gynecology	23.25	22.47	22.21	22.34	22.82	22.64	22.46	22.37	22.2	22.35	21.8	21.26	21.37	21.35	20.85	20.77	−11.717**
	Infectious disease	21.22	18.87	20.4	16.81	20.24	20.13	24.79	18.7	24.68	19.21	20.91	17.84	26.53	22.31	23.18	19.66	10.493**
	Respiratory medicine	52.29	49.26	46.22	48.18	50.24	48.31	44.48	45.99	49.47	45.19	42.36	45.86	50.29	46.87	43.23	45.21	−19.759**
	Stomatology	53.64	52.74	53.32	54	53.16	51.93	52.51	52.67	53.76	52.35	51.43	49.76	49.65	50.68	49.35	48.96	−13.615**
	Internal medicine	6.12	5.85	5.8	5.75	5.55	5.37	5.82	5.56	5.7	5.27	5.51	5.39	5.83	5.4	5.51	5.42	−16.503**
	Surgery	24.58	24.58	24.55	23.72	23.76	23.9	23.72	22.5	22.1	21.99	22.14	21.39	21.4	21	21	20.67	−37.848**
	Other	8.92	8.42	8.51	8.86	8.18	8.15	8.95	9	10.54	9.56	9.31	9.56	11.01	9.43	8.66	9.49	16.714**
**Emergency prescriptions**	
	Emergency internal medicine	40.45	38.6	40.08	39.41	43.87	42.71	44.5	41.78	46.58	43.75	42.54	42.83	49.28	45.59	44.18	42.92	33.050**
	Emergency surgery	34.11	35.81	38.61	36.66	35.39	37.66	36.89	37.02	37.77	37.91	37.41	44.62	47.53	45.62	44.98	46.29	33.521**
**Inpatient records**	
	Pediatrics	39.39	39.5	38.05	43.01	41.36	40.9	38.94	41.42	39.74	41.4	39.96	52.47	51.01	52.4	49.56	51.46	47.203**
	Obstetrics and gynecology	36.28	36.55	36.32	37.06	36.17	36.42	36.14	36.27	35.51	36.35	35.94	41.11	41.11	41.29	40.58	39.74	18.951**
	Infectious disease	35.27	34.97	34.91	33.7	36.16	36.26	37.8	36.45	38.08	36.22	36.77	39.02	42.25	43.3	42.7	40.54	20.799**
	Respiratory medicine	52.49	52.34	52.69	54.33	54.96	53.31	52.45	51.43	52.91	52.12	52.74	60.11	61.32	59.27	58.58	57.73	28.821**
	Stomatology	45.82	44.76	49.58	49.61	50.74	51.36	54.7	51.8	52.28	52.4	56.83	63.4	64.98	64.86	65.47	61.51	24.124**
	Internal medicine	15.53	14.45	14.81	14.84	15.11	14.38	14.63	14.16	15.79	14.15	14.95	16.22	17.82	16.39	16.56	16.19	34.522**
	Surgery	30.89	30.95	31.8	32.13	31.39	31.22	32.14	31.04	32.28	32.76	34.23	38.61	39.08	38.81	39.04	38.03	84.067**
	ICU	27.41	26.83	27.77	28.88	28.01	30.29	31.45	30.42	31.35	30.42	31.15	36.02	38.93	36.96	36.48	35.11	36.336**
	Other	12.95	11.6	12.03	12.16	12.89	11.98	12.29	12.17	13.78	11.84	12.37	16.11	18.97	16.41	16.03	16.01	37.138**

a The Cochran-Armitage trend test was applied to calculate *p* values.

**TABLE 3:** Changes in proportion of prescribed with muti-antibiotics in different clinical departments, 2016–19.

Care type	Clinical department	16Q1	16Q2	16Q3	16Q4	17Q1	17Q2	17Q3	17Q4	18Q1	18Q2	18Q3	18Q4	19Q1	19Q2	19Q3	19Q4	Z^a^
**Outpatient prescriptions**	
	Pediatrics	6.15	5.94	5.76	6.33	6.25	5.78	5.23	5.39	5.56	5.33	4.98	5.78	5.92	5.92	5.98	5.33	-3.812^**^
	Obstetrics and gynecology	13.23	12.51	12.62	13.53	14.18	14.54	14.92	15.41	14.78	15.05	15.17	13.93	12.75	12.22	12.98	12.88	-0.706
	Infectious disease	15.37	17.74	13.78	13.89	15.15	17.4	16.21	16.34	13.35	13.79	13.93	14.81	12.14	13.95	14.7	15.02	-4.391^**^
	Respiratory medicine	10.01	10.43	9.31	8.45	9.04	8.52	8.76	7.28	7.24	7.52	6.98	6.12	7.22	7.59	7.67	7.7	-16.134^**^
	Stomatology	38.46	36.92	37.54	36.98	36.66	36.45	37.68	38.65	38.94	37.87	38.84	37.1	38.08	37.67	38.04	38.33	1.952
	Internal medicine	14.75	15.94	15.66	16.51	15.89	15.78	15.94	16.64	14.63	16.35	17.55	18.25	16.63	17.59	18.76	19.64	19.379^**^
	Surgery	7.48	7.58	7.38	7.28	6.59	6.99	7.1	6.16	6.1	6.65	7.12	6.31	6.15	5.88	6.19	5.86	-11.327^**^
	Other	8.37	9.5	10.05	8	7.99	8.63	9.43	7.76	8.07	8.55	8.67	8.01	7.68	7.98	8.59	7.96	-3.945^**^
**Emergency prescriptions**	
	Emergency internal medicine	16.77	18.06	18.94	17.75	18.6	19.51	18.65	17.8	18.32	19.15	19.52	19.17	18.64	19.18	19.84	18.57	6.765^**^
	Emergency surgery	23.86	23.57	22.93	23.94	26.56	25.23	23.43	22.42	23.3	24.77	23.4	23.64	22.86	23.32	22.32	21.66	-3.624^**^
**Inpatient records**	
	Pediatrics	17.71	17.57	19.1	19.37	18.09	17.6	17.55	18.45	16.94	17	19.34	21.13	19.52	19.28	21.37	19.9	7.345^**^
	Obstetrics and gynecology	28.11	28.33	29.85	29.15	29.42	29.28	28.89	28.32	27.48	28.5	28.32	31.44	30.77	30.98	29.93	29.82	4.508^**^
	Infectious disease	29.37	29.64	29.79	27.11	29.76	29.36	29.01	28.49	30.35	29.04	28.69	29.54	28.87	30.26	29.47	26.96	-0.788
	Respiratory medicine	25.41	25.6	26.05	25.28	26.15	25.37	25.24	24.86	27.27	25.52	25.55	26.38	28.65	27.11	27.36	26.17	6.318^**^
	Stomatology	25.98	30.1	25.9	27.54	28.25	29.37	25.61	29.21	26.55	27.49	26.22	30.71	30.73	29.46	26.23	26.39	0.6
	Internal medicine	17.73	18.53	18.86	18.34	18.22	18.57	18.87	17.86	17.7	17.94	18.14	18.65	19.04	18.83	18.82	18.3	2.411^*^
	Surgery	20.3	20.99	20.66	21.03	20.76	20.56	20.4	20.01	20	19.51	19.78	20.01	19.66	19.89	18.88	17.98	-13.240^**^
	ICU	35.91	34.6	36.04	36.43	37.66	36.5	37	35.59	38.63	36.18	35.67	36.61	39.24	37.43	37.41	36.09	2.700^**^
	Other	18.41	17.59	18.51	19.8	19.52	18.49	20.04	18.73	21.31	20.03	20.05	21.75	22.95	22.44	21.58	20.66	9.403^**^

a The Cochran-Armitage trend test was applied to calculate *p* values.

**TABLE 4:** Changes in proportion of prescribed with parenteral antibiotics in different clinical departments, 2016–19.

Care type	Clinical department	16Q1	16Q2	16Q3	16Q4	17Q1	17Q2	17Q3	17Q4	18Q1	18Q2	18Q3	18Q4	19Q1	19Q2	19Q3	19Q4	Z^a^
Outpatient prescriptions	
	Pediatrics	39.84	35.91	34.03	39.21	38.12	35.35	34.52	36.5	35.66	31.8	30.59	35.31	35.01	30.91	31.04	34.15	-7.345^**^
	Obstetrics and gynecology	9.44	9.57	8.53	8.5	8.64	8.61	7.55	7.03	6.41	6.04	6.99	6.19	5.88	5.44	5.41	4.9	-4.508^**^
	Infectious disease	45.83	43.17	43.49	41.06	47.15	45.69	48.07	46.94	42.85	40.44	41.28	41.72	39.51	38.11	40.6	42.3	-0.788
	Respiratory medicine	17.78	14.25	13.61	13.2	14.38	11.76	11.6	9.48	10.08	7.77	7.16	7.33	8.21	7.47	6.16	5.61	-6.318^**^
	Stomatology	10.11	9.98	10.35	9.27	9.74	10.8	10.96	10.55	10.57	11.98	12.61	12.2	11.28	12.63	12.21	12.19	0.6
	Internal medicine	13.32	12.84	12.69	11.92	11.72	12.15	12.54	11.04	10.27	10.25	9.85	8.88	9.99	9.95	9.91	9.66	-2.411^*^
	Surgery	13.86	14.06	14.19	12.69	11.76	11.83	11.59	9.87	8.39	9.24	9.53	7.94	8.05	8.54	9.12	8.12	-13.240^**^
	Other	7.22	5.98	6.43	5.4	6.01	6.18	6.38	4.43	9.13	8.05	7.52	6.73	8.95	8.1	7.3	7.49	9.403^**^
Emergency prescriptions	
	Emergency internal medicine	67.47	65.63	68.43	67.8	69.91	68.04	68.82	67.85	69.05	68.13	69.93	68.94	69.16	68.88	70.85	70.83	11.738^**^
	Emergency surgery	79.85	78.57	76.52	77.76	80.38	78.49	77.65	81.4	83.04	80.41	78.18	78.52	82.13	80.6	78.18	79.64	3.384^**^
Inpatient records	
	Pediatrics	95.15	94.76	94.82	95.33	95.59	95.65	95.62	95.64	95.62	95.48	95.58	95.86	95.39	95.3	94.79	95.05	0.398
	Obstetrics and gynecology	91.68	91.62	91.73	92.82	91.73	91.57	92.05	92.07	93.09	92.86	92.62	92.57	92.29	92.85	92.35	91.86	3.258^**^
	Infectious disease	91.1	91.19	91.42	91.56	91.77	91.36	90.7	91.3	90.66	89.91	89.08	88.8	89.24	90.05	89.61	89.61	-6.427^**^
	Respiratory medicine	94.58	94.6	94.66	94.72	94.56	94.37	94.63	94.67	93.97	94.03	94.02	94.34	94.43	94.38	94.07	94.23	-3.244^**^
	Stomatology	95.39	94.93	96.53	95.86	95.33	95.26	96.13	96.53	96.72	97.15	96.74	95.24	95.95	95.32	95.27	94.64	-0.804
	Internal medicine	90.05	89.65	89.48	89.65	89.96	89.67	89.61	89.88	89.97	89.95	89.34	89.52	89.99	89.75	89.63	89.61	-0.762
	Surgery	93.88	94.13	94.17	94.17	94.25	94.08	93.95	94.28	94.35	94.43	94.4	94.26	94.42	94.29	94	93.95	1.309
	ICU	95.83	96.46	96.07	96.1	95.94	95.87	95.77	95.95	95.64	96.12	97.03	96.53	96.31	96.38	97.07	95.37	1.595
	Other	89.82	89.21	89.88	90.03	90.15	89.29	90.07	89.63	88.86	88.25	88.37	88.32	89.16	87.78	88.21	88.36	-5.880^**^

a The Cochran-Armitage trend test was applied to calculate *p* values.

**FIGURE 5:**
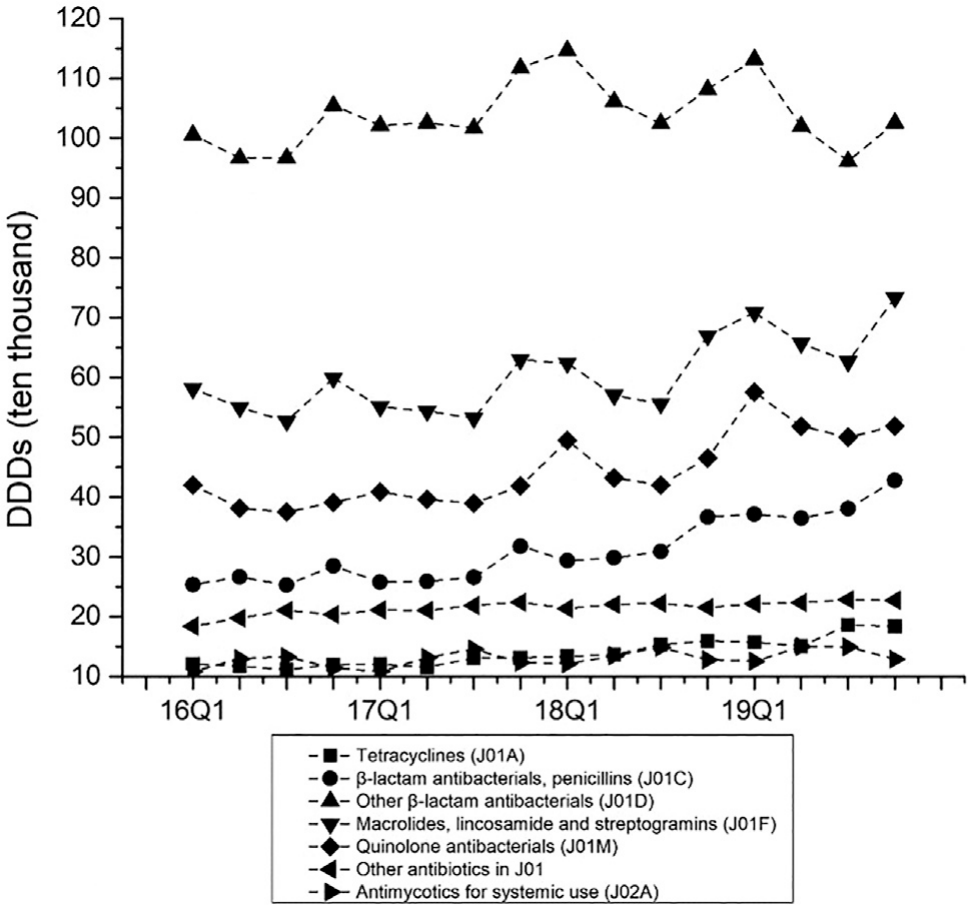
Changes in antibiotics use by ATC classification, 2016–19.

As shown in Figure 5, we found that the proportion of treated with antibiotics, the proportion fo prescribed with muti-antibiotics and the proportion of prescribed with parenteral antibiotics for male patients (*p* < 0.01) and female patients (*p* < 0.01) in emergency departments increased significantly from 2016Q1 to 2019Q4. There was the opposite change for outpatients and inpatients among male patients and female patients. As for the antibiotics use by age group, the overall proportion of treated with antibiotics for patients 5–17 years age (*p* < 0.01), 18–49 years age (*p* < 0.01), 50–64 years age (*p* < 0.01) and ≥65 years age (*p* < 0.01) in outpatient departments decreased over time. The overall proportion of treated with antibiotics for patients <5 years age (*p* < 0.01), 18–49 years age (*p* < 0.01), 50–64 years age (*p* < 0.01) and ≥65 years age (*p* < 0.01) in emergency departments showed an increasing trend over time, which was similar with he overall proportion of treated with antibiotics for all age group of patients in inpatient departments (*p* < 0.01) (Table 5). The overall proportion of prescribed with muti-antibiotics for patients 18–49 years age (*p* < 0.01) in emergency departments showed an upward trend over time, which was contrast to <5 years age (*p* < 0.01), 5–17 years age (*p* < 0.01) and ≥65 years age (*p* < 0.01). The overall proportion of prescribed with muti-antibiotics for patients <5 years age (*p* < 0.01), 5–17 years age (*p* < 0.01) and 18–49 years age (*p* < 0.01) in emergency departments increased over time. For outpatients, the overall proportion of prescribed with muti-antibiotics for patients 50–64 years age (*p* < 0.01) in emergency departments showed an downward trend over time, which was contrast to <5 years age (*p* < 0.01) and 5–17 years age (*p* < 0.01) (Table 6). The overall proportion of prescribed with parenteral antibiotics for all age groups of patients showed a downward trend over time, which was contrast to patients in emergency departments. No significant change was observed in the proportion of prescribed with parenteral antibiotics among all age groups in inpatient departments (Table 7).

**TABLE 5:** Changes in proportion of treated with antibiotics by patient characteristics, 2016–19.

Care type	Patient characteristics	16Q1	16Q2	16Q3	16Q4	17Q1	17Q2	17Q3	17Q4	18Q1	18Q2	18Q3	18Q4	19Q1	19Q2	19Q3	19Q4	Z^a^
**Outpatient prescriptions**	
	**Patient gender**
	Male	14.82	14.21	13.35	14.50	13.85	13.92	13.79	14.62	14.32	13.65	13.02	13.84	14.85	13.96	13.02	13.81	-12.461**
	Female	14.64	13.81	13.21	14.27	13.71	13.73	13.64	14.52	14.39	13.57	13.02	13.87	14.84	13.84	12.91	14.02	-5.881**
	**Patient age**
	<5 years	39.71	39.14	35.16	41.97	37.07	38.54	35.22	40.91	36.55	39.82	35.59	40.83	37.66	39.24	36.30	39.99	-0.633
	5–17 years	36.85	36.43	27.86	41.43	34.20	37.80	28.13	40.81	34.09	35.46	27.12	38.93	35.25	37.08	28.25	38.70	-3.696**
	18–49 years	18.18	17.19	17.01	17.12	17.33	17.00	17.37	17.14	18.00	16.88	16.64	16.77	18.27	17.02	16.46	16.72	-10.145**
	50–64 years	10.27	9.74	9.57	9.66	9.79	9.52	9.73	9.50	10.37	9.21	9.25	9.15	10.34	9.33	9.03	9.06	-13.578**
	≥65 years	8.53	7.79	7.63	7.85	8.01	7.64	7.81	7.80	8.49	7.46	7.29	7.31	8.39	7.43	7.07	7.26	-16.611**
**Emergency prescriptions**	
	**Patient gender**
	Male	38.29	37.17	39.16	37.87	41.20	40.83	42.35	39.59	44.54	41.60	40.58	42.49	47.73	44.46	43.66	42.19	31.365**
	Female	40.34	39.02	40.46	40.02	43.63	42.70	43.65	42.19	45.88	43.78	42.52	43.77	50.31	46.76	45.02	44.73	31.545**
	**Patient age**
	<5 years	31.38	36.05	30.63	34.01	34.01	36.59	33.94	36.49	28.24	33.83	30.40	32.14	38.18	39.54	40.08	32.22	2.625**
	5–17 years	45.35	44.68	41.79	46.33	47.28	44.73	40.89	46.56	43.19	43.21	37.88	43.70	45.98	45.52	44.17	45.55	-0.421
	18–49 years	45.43	44.04	45.51	45.47	47.81	46.95	47.67	46.46	50.63	47.89	47.12	49.54	54.06	50.86	50.05	50.24	27.829**
	50–64 years	35.34	33.63	35.61	34.36	37.93	37.66	39.86	36.77	42.49	38.62	37.65	39.07	46.33	42.26	40.29	39.54	23.703**
	≥65 years	34.83	32.58	35.11	34.00	39.35	38.01	39.66	36.71	42.44	39.60	38.35	39.38	45.66	41.26	39.89	38.60	26.900**
**Inpatient records**	
	**Patient gender**
	Male	23.90	23.20	23.73	24.16	24.02	23.46	23.92	23.29	24.64	23.67	24.73	28.14	29.54	28.24	28.18	27.69	83.270**
	Female	23.73	22.82	23.30	23.83	23.78	22.91	23.17	22.77	24.22	23.08	23.77	26.95	28.35	27.03	26.96	26.72	63.077**
	**Patient age**
	<5 years	39.36	38.58	37.61	41.81	40.70	40.06	38.95	40.93	40.48	41.85	40.00	48.44	47.41	48.01	44.91	47.51	31.012**
	5–17 years	35.65	35.66	36.36	38.84	37.69	37.25	36.66	37.43	36.02	36.40	37.58	47.15	46.75	48.41	44.78	46.46	32.678**
	18–49 years	26.08	26.31	26.20	27.16	26.23	26.17	26.59	25.98	26.27	26.46	27.25	30.98	31.62	31.07	31.12	30.36	54.780**
	50–64 years	20.82	20.27	21.02	21.06	20.76	20.50	20.71	20.18	21.21	20.82	21.84	23.98	24.84	24.03	24.27	23.70	14.132**
	≥65 years	22.58	21.02	21.53	21.74	22.58	21.26	21.59	21.01	23.72	21.37	22.09	25.38	28.04	25.85	25.56	25.28	65.867**

a The Cochran-Armitage trend test was applied to calculate *p* values.

**TABLE 6:** Changes in proportion of prescribed with muti-antibiotics by patient characteristics, 2016–19. prescribed with parenteral antibiotics (c).

Care type	Patient characteristics	16Q1	16Q2	16Q3	16Q4	17Q1	17Q2	17Q3	17Q4	18Q1	18Q2	18Q3	18Q4	19Q1	19Q2	19Q3	19Q4	Z^a^
**Outpatient prescriptions**	
	**Patient gender**
	Male	11.76	12.07	12.30	11.74	11.84	11.78	12.47	11.12	11.12	11.70	12.46	11.53	11.45	11.83	12.56	11.89	-0.088
	Female	12.00	12.60	12.77	12.18	12.46	12.39	13.10	12.05	11.54	12.24	13.14	12.04	11.40	11.95	13.23	12.21	-1.651
	**Patient age**
	<5 years	5.67	5.74	5.21	5.96	5.70	5.12	4.55	5.03	5.15	5.05	4.35	5.25	5.49	5.27	5.29	4.82	-4.037^**^
	5–17 years	6.69	6.34	6.14	6.60	6.81	6.51	6.19	6.13	5.89	5.72	5.69	6.29	6.12	6.37	6.28	5.81	-3.273^**^
	18–49 years	13.90	14.30	14.60	14.49	14.21	14.49	15.09	14.61	13.39	14.44	15.53	14.73	13.63	14.28	15.64	15.41	5.609^**^
	50–64 years	14.44	15.29	14.80	14.83	14.68	14.58	15.34	14.42	13.27	14.71	15.28	14.26	13.36	14.70	15.56	15.36	-0.097
	≥65 years	11.81	12.52	12.00	11.55	11.73	12.04	12.30	11.49	11.21	11.72	11.76	11.20	11.51	11.64	11.50	11.54	-3.263^**^
**Emergency prescriptions**	
	**Patient gender**
	Male	18.68	19.62	20.28	19.43	20.63	21.03	20.34	19.27	20.03	20.79	20.60	20.43	20.14	20.46	20.38	19.59	2.110^*^
	Female	17.09	18.52	19.13	18.16	18.89	19.94	18.57	17.97	17.94	19.38	19.84	19.45	18.39	19.26	20.20	18.61	4.162^**^
	**Patient age**
	<5 years	6.11	7.40	5.26	5.98	3.61	5.39	5.88	3.98	4.03	5.84	6.13	5.76	8.54	12.97	10.58	14.04	6.836^**^
	5–17 years	9.94	12.02	13.13	9.77	10.62	12.07	11.95	10.22	11.01	12.03	13.19	11.88	12.57	13.86	14.43	11.58	3.285^**^
	18–49 years	14.66	16.71	17.28	15.97	17.08	17.51	16.89	16.34	15.59	17.52	18.32	17.37	16.00	16.96	18.08	16.60	3.709^**^
	50–64 years	18.55	20.11	21.38	20.54	21.05	22.25	20.40	20.52	19.43	21.40	20.74	21.36	20.03	20.59	20.84	19.60	0.098
	≥65 years	24.43	25.49	25.05	24.80	25.02	26.84	25.07	23.67	24.67	25.64	24.88	25.49	24.64	25.58	24.84	23.81	-1.056
**Inpatient records**	
	**Patient gender**
	Male	21.37	21.80	22.05	21.84	21.93	21.78	21.81	21.41	21.97	21.25	21.46	22.11	22.43	22.24	21.84	21.08	0.435
	Female	21.23	21.81	22.23	22.13	22.00	21.71	21.81	21.07	21.17	21.04	21.30	22.40	22.59	22.21	21.89	20.99	0.143
	**Patient age**
	<5 years	15.40	15.26	15.84	15.78	14.98	14.84	15.07	15.30	14.69	14.84	16.25	17.32	16.37	16.43	17.70	16.70	5.425^**^
	5–17 years	19.50	20.48	19.64	24.48	21.80	22.01	18.70	23.13	19.91	21.17	20.66	25.66	22.13	23.05	21.33	22.47	4.318^**^
	18–49 years	22.39	23.04	23.31	23.64	23.36	23.42	22.97	23.09	22.59	22.51	22.58	23.87	23.90	23.58	22.71	22.44	-0.17
	50–64 years	22.15	22.52	22.95	22.64	22.96	22.31	22.63	21.69	22.10	21.48	21.82	22.46	22.77	22.47	22.15	20.94	-4.258^**^
	≥65 years	21.01	21.43	21.68	21.04	21.39	21.16	21.65	20.42	21.79	20.92	20.95	21.41	22.35	21.89	21.63	20.71	1.739

a The Cochran-Armitage trend test was applied to calculate *p* values.

**TABLE 7:** Changes in proportion of prescribed with parenteral antibiotics by patient characteristics, 2016–19.

Care type	Patient characteristics	16Q1	16Q2	16Q3	16Q4	17Q1	17Q2	17Q3	17Q4	18Q1	18Q2	18Q3	18Q4	19Q1	19Q2	19Q3	19Q4	Z^a^
Outpatient prescriptions	
	Patient gender	
	Male	20.97	19.01	17.68	19.79	18.53	18.43	17.93	18.67	16.80	16.15	14.97	16.52	16.73	16.04	15.29	16.99	-31.456^**^
	Female	18.38	16.58	15.27	17.06	16.50	15.91	15.24	16.00	14.60	13.34	12.75	14.20	14.63	13.46	12.69	14.25	-35.986^**^
	Patient age
	<5 years	38.76	34.83	31.26	37.31	36.26	33.78	32.12	34.94	35.15	30.91	27.80	33.82	34.83	28.66	28.09	31.95	-19.134^**^
	5–17 years	34.80	31.50	27.93	35.49	33.01	31.22	28.07	33.12	29.77	27.51	25.84	31.55	29.52	28.94	26.58	31.56	-12.961^**^
	18–49 years	14.64	13.67	14.08	12.75	13.10	12.98	13.48	11.51	11.26	10.64	11.01	9.62	10.62	10.43	10.78	9.80	-38.410^**^
	50–64 years	14.40	12.79	12.82	11.45	12.50	11.83	12.89	10.42	11.21	9.82	10.15	8.82	10.51	9.47	9.62	9.04	-27.070^**^
	≥65 years	14.42	12.63	12.16	11.34	12.58	11.99	12.92	10.08	11.39	10.08	10.21	9.14	10.99	10.26	10.18	9.30	-20.658^**^
Emergency prescriptions	
	Patient gender	
	Male	69.95	68.54	70.49	70.03	71.80	70.12	70.68	70.47	71.62	70.95	71.52	70.98	71.67	71.49	72.66	73.24	9.238^**^
	Female	68.87	67.47	69.48	68.94	71.04	69.54	69.97	69.98	70.13	69.38	71.31	70.17	70.35	70.11	71.68	71.44	7.607^**^
	Patient age
	<5 years	21.09	18.70	16.34	14.35	11.81	14.67	14.35	11.23	11.28	10.58	13.66	13.98	22.62	24.06	21.17	23.05	3.396^**^
	5–17 years	41.14	39.20	42.00	38.02	38.92	39.17	41.74	37.66	39.88	37.20	40.82	38.29	40.58	41.65	43.16	43.48	2.299^*^
	18–49 years	59.81	61.21	63.27	61.43	62.37	61.96	62.45	62.50	59.92	61.98	64.02	62.64	59.75	61.81	64.13	63.34	3.780^**^
	50–64 years	74.88	75.03	75.41	76.99	77.77	76.61	76.19	78.26	76.56	77.58	77.22	77.81	77.53	78.49	77.87	78.01	5.910^**^
	≥65 years	88.31	87.17	86.57	87.60	88.57	87.97	86.87	87.99	87.67	88.03	87.39	88.25	88.53	88.39	88.14	88.36	2.854^**^
Inpatient records	
	Patient gender	
	Male	92.74	92.82	92.84	93.02	93.01	92.87	92.75	93.13	92.93	92.95	92.80	92.88	92.96	92.88	92.66	92.67	-1.19
	Female	92.25	92.08	92.09	92.38	92.35	92.06	92.22	92.28	92.27	92.44	92.16	92.21	92.34	92.27	92.04	91.95	-0.88
	Patient age
	<5 years	95.00	94.58	94.89	95.36	95.67	95.12	95.29	95.39	95.30	95.42	95.90	95.71	95.43	94.98	94.87	94.73	0.41
	5–17 years	92.93	92.93	92.84	93.24	93.09	93.33	92.81	93.45	92.94	93.68	92.77	93.43	92.77	93.18	92.45	92.25	-1.343
	18–49 years	91.94	92.04	92.08	92.33	92.15	91.95	92.05	92.16	92.26	92.37	92.14	91.98	91.97	92.01	91.88	91.61	-1.924
	50–64 years	92.34	92.41	92.45	92.40	92.37	92.42	92.40	92.66	92.53	92.62	92.32	92.27	92.52	92.33	92.32	92.19	-0.88
	≥65 years	92.64	92.52	92.53	92.74	92.85	92.49	92.52	92.71	92.54	92.54	92.45	92.60	92.84	92.80	92.47	92.58	0.105

a The Cochran-Armitage trend test was applied to calculate *p* values.

### Antibiotics Category Patterns

**FIGURE 6:**
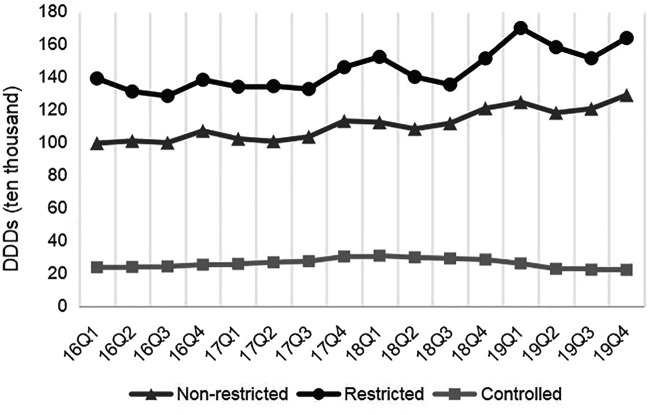
Changes in antibiotics use by different usage levels, 2016–19.

Figure 5 presents the quarterly average consumption of antibiotics classes. The consumption amounts of Tetracyclines (*p* < 0.01), β-lactam antibacterials, penicillins (*p* < 0.01), Macrolides, lincosamide and streptogramins (*p* < 0.01), Quinolone antibacterials (*p* < 0.01), Other antibiotics in J01 (*p* < 0.01), and Antimycotics for systemic use (*p* = 0.03) increased each quarter over the study period. The consumption amounts of restricted antibiotics (*p* < 0.01) and non-restricted antibiotics (*p* < 0.01) increased over time (Figure 6).

**FIGURE 7:**
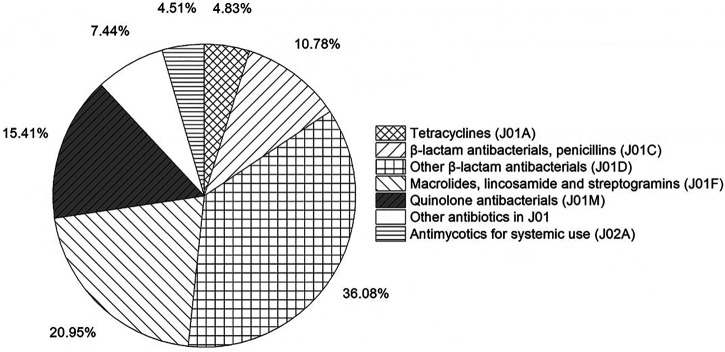
Percentage of antibiotic classes used by ATC classification, 2016–19.

Figure 7 shows the proportion of antibiotics classes from 2016 to 2019. Other β-lactam antibacterials (1,663.03 ten thousand DDD, 36.08%) far exceeded other antibiotics categories. Stablely ranked first, followed by Macrolides, lincosamide and streptogramins (965.74 ten thousand DDD, 20.95%), Quinolone antibacterials (710.42 ten thousand DDD, 15.41%), and β-lactam antibacterials, penicillins (497.01 ten thousand DDD, 10.78%). In terms of individual medicines, the most frequently used antibiotics were levofloxacin (J01MA12), followed by clarithromycin (J01FA09), cefuroxime (J01DC02), amoxicillin (J01CA04) and azithromycin (J01FA10). The proportion of restricted antibiotics, non-restricted antibiotics and controlled antibiotics was 50.00, 38.41 and 9.04%, respectively (Table 8).

**TABLE 8:** Percentage of antibiotic classes used by different usage levels, 2016–19.

Usage level	DDDs (ten thousand)	Usage ratio (%)
Restricted	2,304.87	50.00
Non-restricted	1770.74	38.41
Controlled	416.86	9.04

## Discussion

Based on the prescribing data from the CPDHP during 2016–19, this multi-center study analyzed 3,422,710 outpatient and emergency prescriptions and 26, 118, 436 inpatient records from 89 tertiary hospitals in nine cities. Results showed that the reasonable use of antibiotics in outpatient and emergency departments was superior to inpatient departments. The overall status of antibiotics use for inpatients in China rebounded during 2016–19 is noteworthy, showing a significant upward trend in treating with antibiotics. A close association exists between AMR and the amount of antibiotics used (Bell et al., 2014), which indicates a severe need to promote antibiotic consumption. Furthermore, the antibiotics use varied by different survey areas, clinical departments, patient gender, patient age and antibiotics categories.

Because the sampled 89 hospitals were from nine different administrative provinces, there may be inter-drug supply variation resulting from differences in various drug procurement policy. However, the preliminary test in our study demonstrated that there were no obvious differences in antibiotics consumption and cost in nine cities. As a consequence, differences in drug supply varieties, specifications and prices in different cities may have slight influence on the results in this study. One possible reason is that most of the surveyed antibiotics were included in the National Reimbursement Drug List (NRDL) or Chinese National Essential Medicine List (NEML). The prices and dosage forms or specifications of surveyed antibiotics are set in a relatively uniform level. WHO recommended that the proportion of treated with antibiotics should not be over 30% (WHO, 2006). Except for emergency departments, the percentage use of antibiotics in the current study (16.05% for outpatients, 25.19% for inpatients) was lower than this recommended level and lower than in many other countries. For example, the average proportion of prescribed antibiotics in some developing countries was 40–50%. The proportion of prescribed antibiotics for outpatients was 60–70% in some low-and-middle-income countries such as Pakistan, Indonesia and Mozambique (WHO, 2004; WHO, 2009).

The percentage of antibiotics use under effective control may be related to a series of antimicrobial administration policies adopted by the Chinese government. China has developed Antimicrobial Stewardship Programs (ASPs) to promote antibiotics use. It’s important to note that a part of the provinces has implemented restrictions of antimicrobial use for outpatient in recent years. Previous studies also revealed that antimicrobial stewardship could reduce the consumption of antibiotics (Hou et al., 2014; Uda et al., 2019; Walker et al., 2019).

Several issues are still noteworthy even though the use of antibiotics in China has been controlled to some extent. First, the average proportion of treated with antibiotics in Stomatology was high (over 50%). More than one-third of dental patients received antibiotic combination treatment, and the percentage of prescribing parenteral antibiotics increased with time. Second, the percentage of antibiotics used for adults (>18 years) increased during the study period is noteworthy. The percentage of prescribing parenteral antibiotics for patients 5–17 years age and 18–49 years of age exceeded 50%. This finding may be related to young children’s low immunity and the pressure from patients who insist on antibiotics therapy. As the primary breadwinners of a family, young people want to be cured as fast as people in most cases when they fall ill (Lin et al., 2016). Third, co-prescription of at least two antibiotics for patients ≥65 years of age and in ICU may result from the severity and complexity of those patients. Finally, this study revealed that most categories of antibiotics use increased with time.

The above finding suggests that nationwide education and prescription control of antibiotics to treat common but not severe infectious should be reinforced (Kim et al., 2019). In fact, different antibiotic classification catalogs may have different influence on the management of antibiotics use in health-care institutions. Hence, a unitary classification of antibiotic is urgently needed to make for easier administration. Besides, the medical decision-making surrounding antibiotic use is very complex and the size and types of medical care are varied, which can lead to the flexibility of implementation of ASPs in Chinese urban hospitals. Thus, the institutionalized ASPs should be implemented at the hospital level to improve the utilization of antibiotics. Individual hospitals should implement ASPs, which reflects their environment. To design effective interventions to improve antibiotics use, it is significant to understand patients’ characteristics and obstacles for antibiotics prescribing from physicians or hospitals perspectives (Wang et al., 2016).

There are several limitations to our study. First, due to data collection difficulties, we cannot link antibiotics records with patients medical records or laboratory results. Second, primary health-care institutions and secondary hospitals were not considered in this study, which might not represent the situation of antibiotics consumption comprehensively. Third, our study mainly focused on the patterns of antibiotic use and expenditure. Other factors that may influence our indicators were not considered in the current study, which needed further exploration. Despite these limitations, this study still revealed crucial new information on antibiotics use and how these changes in antibiotic consumption changed in tertiary hospitals from nine cities, providing evidence for policy-makers to improve antibiotic stewardship interventions.

## Conclusion

In conclusion, this study found that the proportion of treated with antibiotics meet the WHO standards, suggesting the ASP was effective in China. This study also demonstrated that the reasonable use of antibiotics in outpatient and emergency departments was superior to inpatient departments. The antibiotics use varied by different survey areas, clinical departments, patient gender, patient age and antibiotics categories. Further research is required to obtain more detailed information on the inappropriate use of antibiotics in China. To better facilitate proper antibiotics use, the next stage of policy should focus on improving the appropriateness of antibiotics uses at the individual level.

## Data Availability

The original contributions presented in the study are included in the article/supplementary material, further inquiries can be directed to the corresponding authors.
